# Salivary miRNAs Expression in Potentially Malignant Disorders of the Oral Mucosa and Oral Squamous Cell Carcinoma: A Pilot Study on miR-21, miR-27b, and miR-181b

**DOI:** 10.3390/cancers15010291

**Published:** 2022-12-31

**Authors:** Dario Di Stasio, Antonio Romano, Ciro Emiliano Boschetti, Marco Montella, Laura Mosca, Alberta Lucchese

**Affiliations:** 1 Multidisciplinary Department of Medical Surgical and Dental Specialties, University of Campania “Luigi Vanvitelli”, 80138 Naples, Italy; 2 Depatment of Hygiene and Mental Health, University of Campania “Luigi Vanvitelli”, 80138 Naples, Italy; 3 Department of Advanced Medical and Surgical Sciences, University of Campania “Luigi Vanvitelli”, 80138 Naples, Italy

**Keywords:** oral potentially malignant disorders, oral squamous cell carcinoma, oral epithelial dysplasia, biomarkers, early diagnosis, miRNAs, miR-21, miR-27b, miR-181b, mouth diseases, precancerous conditions

## Abstract

**Simple Summary:**

Management of potentially malignant oral disorders pose a challenge to the clinician. These lesions, over time, can undergo cellular and morphologic alteration (dysplasia) until they evolve into squamous cell carcinoma. Diagnosis is made by biopsy and histologic analysis. Screening methods that noninvasively allow these lesions to be monitored both diagnostically and prognostically (biomarkers) have long been sought. A class of non-coding RNAs called miRNAs is readily found in biological fluids and in saliva: they fit into this area of research. From the data presented in this study, miR-181 appears to be a promising salivary biomarker for dysplasia.

**Abstract:**

(1) Background: Oral potentially malignant disorders (OPMD) represent a fundamental challenge for clinicians, considering the possibility of progression into oral epithelial dysplasia (OED) and oral squamous cell carcinoma (OSCC). Several studies have examined the expression of miRNAs in humans as diagnostic and prognostic biomarkers. Among these, miR-21, miR-27b, and miR-181b proved to be promising. This cohort study evaluated the different expressions of those miRNAs in the saliva of patients with OPMD and OSCC. (2) Methods: Patients with a clinical diagnosis of OPMD and/or OSCC were enrolled; saliva samples were collected; miRNAs were extracted and quantified via qRT-PCR was performed. Data were analyzed by subgroups based on the histopathological diagnosis (OSCC and the grade of OED) using the ΔΔCt method. Saliva from 10 healthy donors was used as the control. One-way ANOVA and Kruskal–Wallis tests were performed to assess the differences between groups. (3) Results: 23 patients for the OPMD group (6 with no dysplasia, 7 with low-grade, and 10 with high-grade dysplasia) and 10 with OSCC were analyzed. MiR-21 did not show any variation among groups; miR-27b was under-expressed in dysplastic lesions (*p* = 0.046); miR-181b was upregulated in high-grade dysplasia (*p* = 0.006), increasing with the degree of dysplasia, and decreasing in OSCCs. (4) Conclusions: Salivary miR-27b and miR-181b could be promising biomarkers for oral dysplasia. Further studies are needed to clarify their feasibility.

## 1. Introduction

Saliva represents a biological fluid rich in electrolytes, hormones, proteins, peptides, and nucleic acids, including non-coding RNAs [1]. These non-coding RNAs are usually categorized as long ncRNAs (lncRNAs), small interfering RNAs (siRNAs), and microRNAs (miRNAs). Over 1000 species of miRNAs are decoded by human cells: miRNAs represent double-stranded non-protein coding nucleic entities comprising between 17 and 25 nucleotides, and they can simultaneously regulate the expression of hundreds of genes [2]. Several miRNAs play a critical role in autoimmunity, cell proliferation, differentiation, and cell death [3,4,5]. Hsa-miRNAs are present in all biological fluids, and their expression is essential in maintaining regular cell functions [4].

Several studies have shown that miRNAs in saliva, wrapped up by proteins or stored within exosomes, are stable and can resist degradation. MiRNAs could enter saliva from the blood through various cellular mechanisms, such as transcellular or paracellular ways, or they could also be produced locally by cellular apoptosis or necrosis and could also be released from normal epithelial or cancer cells in exosomes. Profiling of miRNAs isolated from exosomes and their donor cells indicates that exosomes are highly enriched in RNA, as well as miRNAs, which reflect their cellular expression [6,7].

In contrast, miRNA dysregulation may be associated with the pathogenesis of inflammatory and malignant diseases [8]. Repression of these regulatory targets leads to a depression of the translational efficacy of mRNA [9]. MiRNAs may not only regulate the expression of oncogenes and tumor suppressor genes from cancer progression but may also function directly as oncogenes or tumor suppressor genes in carcinogenesis [10]. Hence, reduced miRNA expression is involved in the progression of squamous carcinoma (OSCC) [8]. Besides, oral potentially malignant disorders (OPMD) are a fundamental challenge for clinicians, considering the possibility of malignant transformation [11,12]. Leukoplakia, oral lichen planus, oral submucous fibrosis, and erythroplakia represent the most common type of OPMD [13,14]. Some factors have been associated with an increased risk of malignant transformation: sex, site of onset, type of lesion, smoking habit, alcohol consumption, and the presence of epithelial dysplasia (OED) on histologic examination [15,16]. OED is characterized by cellular atypia and loss of normal maturation and stratification [17]. Oral dysplasia is defined, according to the WHO Classification of Tumours, 4th Edition (2017), in a binary system (low-grade dysplasia—LD—and high-grade dysplasia—HD) [18].

OSCC is a type of cancer characterized by a low survival rate [19,20]. This malignancy accounts for more than 200,000 new cancer cases annually worldwide. Despite remarkable advances in diagnosis and therapy, the five-year survival rate of patients with OSCC remains below 50% [21]. Therefore, early diagnosis of oral malignancy can significantly change the 5–10 years survival rate [22]. The TNM staging system used to classify OSCC patients does not adequately report the molecular heterogeneity of those tumors [23]. The necessity to acquire a more detailed molecular characterization and identify biomarkers for the early detection of lesions is suggested [24]. However, although several studies investigated molecules that can potentially predict malignant transformation, response to therapy, or clinical outcome in OSCC, no biomarkers have been introduced in clinical practice [25]. The diagnostic value of dysregulated miRNA signatures in OSCC highlights the possible importance of miRNAs as prognostic markers of malignant transformation in OPMDs [26]. Several studies have examined the expression of miRNAs from human specimens (blood serum/plasma, saliva, tissue) as diagnostic and prognostic biomarkers in patients with OPMD, some of which are used as risk stratification biomarkers for malignant transformation, showing promising findings. Some interesting associations between altered miRNA expression and cytological and histopathological parameters used to grade dysplasia have not yet been investigated [27]. Current data revealing the association of miR-21, miR-27b, and miR-181b in oral potentially malignant progression still show a gap of knowledge about the link between certain carcinogens and salivary miR-21, miR-27b, and miR-181b expression. This study aims to evaluate a specific salivary miRNA model for OPMD and oral squamous cell carcinoma (OSCC).

## 2. Materials and Methods

For this cohort study, the manuscript was prepared and revised according to the STROBE Statement–checklist of items (Supplementary Material Table S1).

### 2.1. Patient Enrolment and Selection

Thirty-six patients were recruited consecutively (from May to December 2021) from the Oral Medicine Division and Internal Medicine Units of the University of Campania “Luigi Vanvitelli.” For the study group, patients of both sexes aged over 18 with a clinical diagnosis of OPMD and/or OSCC have been enrolled. Ten healthy patients of both sexes aged over 18 were enrolled in the control group (a total of 46 subjects).

In both groups, patients with other malignancies (non-OSCC) and/or under treatment with chemo and radiotherapy, patients who have undergone an organ transplant, patients with a positive history of chronic viral diseases, and patients under therapy with systemic or topical oral corticosteroids were excluded. Patients with drug-induced or contact lichenoid reactions and pregnant or lactating women were also excluded. Moreover, all patients enrolled were tested for SARS-CoV-19 infection before sample collection, and we selected only SARS-CoV-19-free patients.

Cigarette and alcohol consumption were not considered among the exclusion criteria. However, patients were asked to suspend for at least one week before being able to do the salivary samples, as well as for antibiotics, NS anti-inflammatory agents, analgesics, or chlorhexidine mouthwashes. The same was required of the participants of the control group.

At the examination, the salivary samples were collected. All patients of the study group underwent histological analysis; they were subsequently divided into the following subgroups based on their histopathological diagnosis (WHO classification, 2017) [18]: (a) OSCC group; (b) OPMD group.

Written informed consent was obtained from all participants. The study was approved by the ethical committee of the University of Campania “Luigi Vanvitelli” (#0000339/2018). Details regarding patients of all groups are reported in Table 1.

### 2.2. Saliva Collection and Storage

The protocol for collecting all saliva samples was developed according to the standards presented in the literature [28]. The saliva samples were collected once, from 08:00 to 12:00 a.m., to minimize variability. The enrolled subjects were welcomed in a quiet and friendly atmosphere. During the collection period, the subject was sitting with eyes open and head slightly tilted forward, reducing the orofacial movements to minimize the influences on salivary flow. Immediately before the start of the collection, the subject was instructed to swallow. The patient allows the saliva to accumulate on the floor of the mouth for 60 s without swallowing. The patient emptied the saliva collected, and the procedure was then repeated four more times for a total collection time of 5 min. The subjects were instructed not to swallow during the collection period of 5 min. Following the collection, saliva was processed within the shortest time possible to avoid cell damage and subsequent miRNA release from exosomes in saliva.

### 2.3. MiRNA Isolation, Amplification, and Detection

The MiRNA extraction was performed using a MIRVANA PARIS KIT (Thermofisher Scientific, Waltham, MA, USA). It was optimal for our purpose because of its higher sensitivity. Subsequently, using the TaqMan^®^ MicroRNA Reverse Transcription Kit (Thermofisher Scientific), single-stranded cDNA was synthesized from total RNA samples, as reported in our previous work [29]. Then, cDNA targets were pre-amplified to increase the quantity of the desired cDNA for gene expression analysis by DNA polymerase from the TaqMan^®^ PreAmp-Master Mix using sequence-specific primers and probes on the Megaplex PreAmp primers. The pre-amplified products were used to evaluate the different expressions of miR-21, miR-27b, and miR-181b by quantitative real-time PCR (qRT-PCR) (Taqman, Life Technologies, Carlsbad, CA, USA) using TaqMan miRNA Assays that use looped-primer to detect mature miRNAs accurately. qRT-PCR was performed on a ViiA7 Real-time PCR system (Applied Biosystems, Foster City, CA, USA). The small-nuclear-U6 was selected as an appropriate constitutively expressed endogenous control to normalize total RNA samples. The transcripts’ relative expression was measured using ViiA7-Real-Time PCR software (Applied Biosystems, Foster City, CA, USA).

### 2.4. Outcomes

The study’s primary outcome is to evaluate the expression of miRNAs miR-21, miR-27b, and miR-181b in subjects with OPMD and OSCC by comparing the data obtained with a control group. Specifically, the following analyses were performed: (1) miRNA expression in the OPMD group; (2) miRNA expression in the OSCC group; (3) OSCC group vs. OPMD group. The secondary outcome aims to analyze, within the OPMD group, the expression of selected miRNAs according to the degree of dysplasia (binary system): high-grade (HG) dysplasia vs. low-grade (LG) dysplasia and no dysplasia (ND).

### 2.5. Data Analysis

Statistical analysis within the study subjects was carried out and compared with the control group. Analyses were performed using IBM^®^ SPSS^®^ Statistics (ver. 28.0.1.0).

In order to assess the statistical power and significance of ΔCt variation in OPMD, OSCC and control groups, the power analysis was performed using G*Power 3.1.9.6 (Heinrich Heine University, Düsseldorf, Germany) with a one-way ANOVA assuming a medium Cohen’s effect size of 0.5 (α = 0.05). Sensibility (statistical power) was fixed at 0.80.

The expression of each miRNA tested was normalized by the ΔΔCt method [5]. The 2-ΔΔCT method has been extensively used as a relative quantification strategy for quantitative real-time polymerase chain reaction (qPCR) data analysis. This method is a convenient way to calculate relative gene expression levels between different samples. It directly uses the threshold cycles (CTs) generated by the qPCR system for calculation.

A database system using IBM^®^ SPSS^®^ Statistics (ver. 28.0.1.0.) was created, and ΔCt difference among groups was calculated using one-way ANOVA. Post hoc tests were used for multiple comparisons. The Kruskal–Wallis test was used to assess the differences within the OPMD group; the Mann–Whitney test was used to test the expression of miRNAs depending on the OSCC grade. The Spearman test was performed to exclude correlation between the independent variables. A receiver operating characteristic (ROC) curve was made to assess the diagnostic ability of the analyzed miRNAs for OED and OSCC. The significance level was considered at *p* ≤ 0.05. When required, a Bonferroni correction has been applied. Graphs were produced with GraphPad Prism (ver. 9.4.1-458).

## 3. Results

A total of 46 patients with a mean age of 60.02 ± 16.22 years (28 males—60.9% and 18 females—39.1%) were enrolled in the study. Three patients (2 with OPMD and 1 with OSCC) were excluded from the study because the quality of the collected salivary samples did not allow analysis. The OPMD group consisted of 23 people (15 males and eight females) with a mean age of 61.22 ± 11.87 years; among these, six patients presented epithelial hyperkeratosis with no dysplasia (ND), seven were diagnosed with low-gradeOED (LG), and 10 with high-grade OED (HG).

Ten patients were enrolled in the OSCC group (six males and four females) with a mean age of 69.90 ± 9.78. Seventy percent of the OSCCs were localized to the tongue (four ulcerated and three nodular), 20% were exophytic lesions at the level of the alveolar ridge, and finally, one ulcerated lesion at the level of the retromolar trigone. Histopathological analysis established the grading of the lesions: 50% were in G1, and 50% were in G2. The expression of miR-21, miR-27b, and miR-181b did not show a significant difference between G1 and G2 lesions (*p* > 0.05).

The control group, consisting of 10 healthy volunteers (six males and four females), had a mean age of 45.20 ± 20.22 years. The age of patients in the control group is significantly lower than in the other groups; however, a correlation between age and expression of miR-21 (*p* = 0.523), miR-27b (*p* = 0.691), and miR-181b (*p* = 0.389) was not found in the present study.

All values inherent in the expression of each miRNA tested in each group were reported in Table 2.

### 3.1. miRNA Expression in the OPMD Group

**Figure 1:**
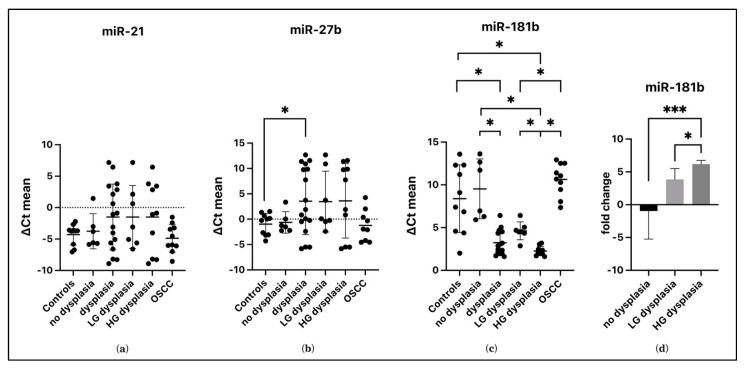
Salivary miRNAs expression in OED, ND, OSCC groups and in Controls. Subgroups HG and LG dysplasia were added. The ΔCt mean of all salivary samples from patients for each miRNA is represented (**a**–**c**). The fold change of miR-181b within the OPMD group is reported (**d**). Asterisk = the comparison between groups, is significant for * *p* ≤ 0.05, *** *p* ≤ 0.001.

**Figure 2:**
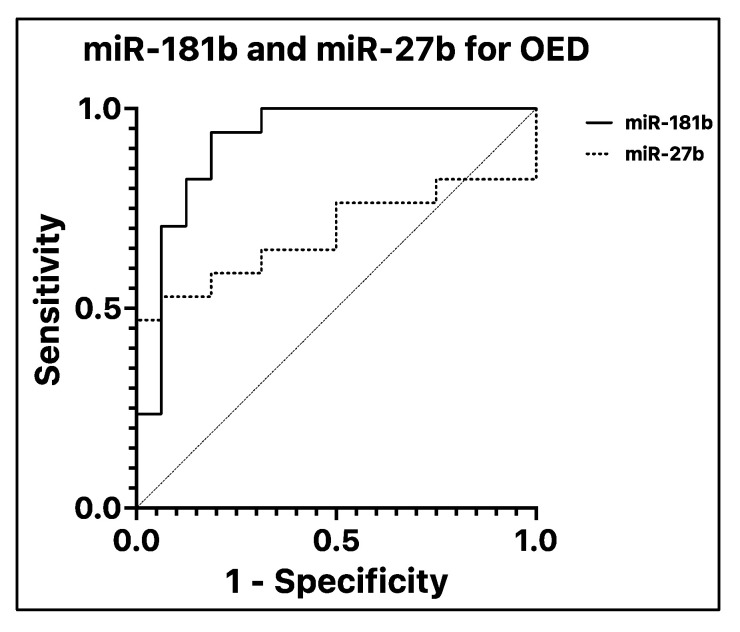
Receiver operating characteristic curve (ROC) and area under the curve (AUC). ROCanalysis of miR-181b (straight line) and miR-27b (dotted line) for discriminating OED from OPMD without dysplasia (ND and control groups). MiR-181b has very good diagnostic values in terms of sensitivity and specificity (AUC = 0.9154; standard error = 0.05315; 95% C.I. = 0.8113 to 1); miR-27b, on the other hand, is below the acceptable threshold (AUC = 0.6875; standard error = 0.09916; 95% C.I. = 0.4931 to 0.8819). The reference line is represented with a dashed line.

No variation in the salivary expression of miR-21 (*p* = 0.122) has been found, while miR-27b reported a variation between groups (*p* = 0.046). The difference between its concentration in patients with dysplastic and non-dysplastic lesions was not significant (*p* = 0.77). However, it was under-expressed in dysplastic lesions by 4.52 folds (*p* = 0.042) compared with controls. The salivary concentration of miR-181b varied among the groups (*p* < 0.001). In particular, there was overexpression in patients with dysplasia compared with controls of 5.14 folds (*p* = 0.006) and non-dysplastic lesions of 6.29 folds (*p* = 0.014). There was no difference in miR-181b expression between controls and non-dysplastic lesions (*p* = 0.717)—Figure 1. The ROC curve analyses revealed that salivary miR-181b presented an area under the curve (AUC) of 0.915—*p* < 0.001 (Figure 2). At the optimal cutoff value of 5.48, the values of sensitivity and specificity were 94.1% and 81.2%, respectively. MiR-27b did not show significant values of AUC (0.688—*p* = 0.66).

### 3.2. miRNAs Expression in OSCC Group

The expression of miR-21 did not change in the different groups (*p* = 0.062). The salivary concentration of miR-27b changed in the groups (*p* = 0.018), but there was no difference between OSCC and non-dysplastic lesions (*p* = 0.863), dysplastic lesions (*p* = 0.051) and control group (*p* = 0.905). MiR-181b was under-expressed in patients with OSCC of 7.39 folds (*p* < 0.001) compared to patients with dysplastic lesions. However, the expression of this miRNA was not significant when comparing non-dysplastic lesions (*p* = 0.891) and controls (*p* = 0.253).

### 3.3. OSCC Group vs. OPMD Group

When the expression of miRNAs is compared among the various subgroups, miR-21 (*p* = 0.197) showed no significant differences (Figure 1a). Salivary miR-27b changed among groups (*p* = 0.032), but multiple comparisons showed no significant differences (*p* > 0.05—Figure 1b).

Salivary concentrations of miR-181b appeared to vary significantly compared with controls. Specifically, in patients with HG dysplasia, miR-181b expression was upregulated, compared with controls, by about 6.1-fold (*p* = 0.006). Moreover, expression of salivary miR-181b increased with increasing degree of dysplasia, discriminating between HG dysplasia and LG dysplasia (2.6 folds difference—*p* = 0.04) and OPMD without dysplasia (7.27 folds difference—*p*< 0.001) as shown in Figure 1d.

The expression of this miRNA in OSCC does not differ from controls (*p* = 0.509) and non-dysplastic lesions (*p* = 0.948). At the same time, it is under-expressed concerning HG dysplasia (8.37 folds difference—*p* < 0.001) and LG dysplasia, as reported in Figure 1c (3.5 folds difference—*p* < 0.001).

## 4. Discussion

The diagnosis of OPMD and subsequent grading of the dysplasia are clinically significant for the patient’s follow-up and management [30]. The current gold standard for identifying dysplasia and assessing cancer risk is the histological analysis of biopsies, following the WHO grading system [18]. The histological approach (especially in small biopsies) is subject to limitations and different interpretations among pathologists. For example, ulcers and inflammatory areas can change the morphological features. Furthermore, the “non-strict” criteria of the WHO classification do not allow a clear distinction between dysplastic lesions (low-grade and high-grade dysplasia), leading to a lack of agreement in assessing the diagnoses [31]. To stratify the cancer risk, identifying several molecular biomarkers, either alone or in combination with histology, represents the main challenge to improving dysplasia diagnosis and steering clinical therapy [32].

In this light, we showed for the first time that miR-181b was overexpressed in patients with dysplastic lesions compared with patients who had OPMD without dysplasia and compared with the control group. Within the OPMD subgroups, based on histological diagnosis, expression of salivary miR-181b increased with an increasing degree of dysplasia, allowing us to considerate miR-181b a promising molecular biomarker for the cancer risk stratification.

MiR-181b has been associated, in ex vivo studies on histopathological samples, with different mechanisms involved in the dysplastic process [26,33]. To our knowledge, this is the first time that miR-181b has been studied as a salivary biomarker in OED. Deregulated expression of these miRNAs has also been previously reported in other cancers (upregulated in B-cell chronic lymphocytic leukemias, brain tumors, thyroid, and gastrointestinal carcinomas) [34].

The presented results highlight the role of miR-181 in OED, although it is based on a small sample of cases. In particular, the increase in architectural and cytological changes (which determine the degree of dysplasia) could coincide with an increased expression of this miRNA in the saliva. In an ex vivo study of the expression of specific miRNAs in dysplastic samples of oral leukoplakia, Brito et al. found increased expression of miR-181b in samples with a higher number of mitotic figures, increased nuclear/cytoplasmic ratio, or hyperchromia [33]. Moreover, Cereivigne et al. found a significant over-expression of miR-181b in both progressive oral leucoplakias and same-site OSCCs, confirming coherent increases in expression levels linked with lesion severity [34]. Also, Avissar et al. detected an increase in miR-181b expression in tumors compared with normal tissue [35]. Unfortunately, in the present study, the expression of salivary miR-181b increases from healthy tissue to HG dysplasia. It then decreases considerably in OSCC, which does not show different levels of upregulation from control. This difference could be explained by the different study designs (in vivo or ex vivo) and different sampling targets (on saliva or tissue). For the authors, the different expression of miR-181b in dysplasia (upregulation) and OSCC could also be the result of the total loss of tissue architecture that occurs in OSCC, with deregulation occurring in a negative feedback mechanism. Further in vivo studies on a larger sample will be needed to elucidate the expression of this miRNA in OSCC. Assao et al., on the other side, in an ex vivo study on tissue samples, observed an upregulation of miR-181b in actinic cheilitis without epithelial dysplasia compared to actinic cheilitis with epithelial dysplasia or lip cancer, suggesting a different behavior for miR-181bin lip epithelium [36].

Regarding miR-27b, data from the present study show an under-expression of this miRNA in dysplastic lesions compared with controls but no difference between this group and the OSCC group. In previous work, this miRNA was found to be down-expressed in the saliva of patients with OLP (which is an OPMD) without dysplasia [29]. Moreover, the data presented also disagrees with another study conducted on saliva by Momen-Heravi et al. Their research showed a progressive overexpression of this miRNA from samples of patients with OLP to those with OSCC [9]. Aghbari et al. compared the expression of miR-27b in tissues and saliva between patients with OLP and controls. They evaluated their use as biomarkers of disease activity and the tendency for malignant transformation [37]. MiR-27b has been associated with human keratinocyte differentiation in vitro and in vivo and regulates the expression of matrix metalloproteinase 13 and the transforming growth factor-beta (TGF-beta)/bone morphogenetic protein signaling, immune response, and chronic inflammation [37,38]. Other studies reported a reduction in the expression of miR-27b in OSCC specimens, assuming this miRNA’s role as a tumor suppressor through targeting the MET oncogene in this disease [39]. This onco-suppressor activity is widely recognized in several in vitro studies, acting on various signal pathways by targeting FZ27 and Wnt [40]. On these premises, further studies are needed to evaluate this miRNA as a salivary biomarker of malignant transformation.

Finally, miR-21 is one of the miRNAs most associated with the mechanisms of malignant transformation of oral lesions. A recent systematic review indicated that miR-21 could provide a prognostic indicator in OSCC [26]. The data presented do not confirm this tendency, but a larger sample of patients could probably give different results.

The main limitation of this pilot study is the sample size. For this type of salivary sampling study and subsequent validation of a biomarker for dysplasia and OSCC, multicenter studies with a larger population will be necessary. In addition, it would be interesting to follow the evolution of the dysplastic lesions’ development at different time points, evaluate the expression of these miRNAs in the eventual malignant transformation at the same oral site, and compare the expression of salivary miRNAs with those on biopsy tissue.

## 5. Conclusions

In conclusion, increasing data shows that miRNAs play an important role in cancer pathogenesis and OED progression [17,21]. Although tumor biopsy represents the gold standard for diagnosis, salivary miRNAs could represent a valid non-invasive biomarker for OED screening and a signature that could help distinguish between the various grade of dysplasia [32]. Data presented in this study show that salivary miR-181b over-expresses in dysplastic lesions (in particular in HG dysplasia), making this miRNA a promising biomarker in OPMDs evaluation. Further studies will be needed to establish its feasibility in clinical practice.

## Figures and Tables

**Table 1:** Demographic data and comorbidities of patients of the three groups.

	Controls	OPMD	OSCC	*p*-Value (2-Tailed*)*
Sex (M/F)	6/4	16/9	6/5	0.865 *
Mean age (±SE)	45.20 ± 6.40	61.96 ± 2.59	69.09 ± 2.91	0.009 **
Cigarette consumption (%)	0.20	0.61	0.36	0.075 **
Alcohol consumption (%)	-	-	-	-
Other comorbidities (%) ***	0.30	0.57	0.81	0.057 *

* Chi-square test; ** Kruskal–Wallis test; *** Hypertension, thyroiditis, type 1 and type 2 diabetes; inflammatory bowel diseases, dyslipidemia.

**Table 2:** miRNAs expression in the analyzed groups. The significance values refer to the comparison of the mean of each group and subgroup with the control group.

			miR-21	miR-27b	miR-181b
Controls		C_T_	17.201	20.515	29.975
		ΔC_T_	−4.283	−0.969	8.373
OPMD	No dysplasia	C_T_	15.619	18.741	28.904
		ΔC_T_	−3.756	−0.633	9.530
		ΔΔC_T_	0.527	0.336	1.157
		Log_2(2-(ΔΔCT))_	−0.527	−0.336	−1.157
		*p*-value (2-tailed)	0.999	0.950	0.971
	Dysplasia	C_T_	23.677	28.726	27.479
		ΔC_T_	−1.491	3.558	3.234
		ΔΔC_T_	2.792	4.527	−5.139
		Log_2(2-(ΔΔCT))_	−2.792	−4.52	5.139
		*p*-value (2-tailed)	0.129	0.042	0.006
	LG-dysplasia	C_T_	22.502	27.465	27.612
		ΔC_T_	−1.494	3.469	4.624
		ΔΔC_T_	2.789	4.438	−3.749
		Log_2(2-(ΔΔCT))_	−2.789	−4.44	3.749
		*p*-value (2-tailed)	0.573	0.405	0.093
	HG-dysplasia	C_T_	24.500	29.609	27.390
		ΔC_T_	−1.489	3.621	2.261
		ΔΔC_T_	2.795	4.590	−6.112
		Log_2(2-(ΔΔCT))_	−2.795	−4.590	6.112
		*p*-value (2-tailed)	0.477	0.373	0.006
OSCC		C_T_	17.212	19.708	31,987
		ΔC_T_	−4.079	−1.220	10,626
		ΔΔC_T_	0.204	−0.251	2253
		Log_2(2-(ΔΔCT))_	−0.204	0.251	−2253
		*p*-value (2-tailed)	0.945	0.997	0.509

## Data Availability

The data presented in this study are available on request from the corresponding author.

## Supplementary Materials

The following supporting information can be downloaded at: https://www.mdpi.com/article/10.3390/cancers15010291/s1, Table S1. STROBE Statement–checklist of items.

## Abbreviations

Oral potentially malignant disorders (OPMD); oral epithelial dysplasia (OED); oral squamous carcinoma (OSCC); high-grade dysplasia (HG); low-grade dysplasia (LG); no dysplasia (ND).
