# Supplementary material for: Salivary miRNAs Expression in Potentially Malignant Disorders of the Oral Mucosa and Oral Squamous Cell Carcinoma: A Pilot Study on miR-21, miR-27b, and miR-181b
Source: Cancers (Basel). 2022 Dec 31;15(1):291. doi: 10.3390/cancers15010291 (PMC9818333; doi:10.3390/cancers15010291)
Supplement: Supplementary file 1 [file cancers-15-00291-s001.zip › cancers-2072872-supplementary.pdf]

Table S1. STROBE Statement–checklist of items.

Patient #	Age	Sex	Group	Smok-ing	Alcohol	Comorbidi-ties	Diagnosis	miR21_dCt	miR27b_dCt	miR181_dCt
1	50.00	M	OSCC	yes	no	yes	OSCC - G1	−5.93	−4.55	11.06
2	63.00	M	OSCC	yes	yes	yes	OSCC - G2	−6.03	−0.32	11.41
3	63.00	F	OSCC	no	no	yes	OSCC - G1	−2.42	2.03	12.53
4	70.00	F	OSCC	no	no	no	OSCC - G1	−3.45	0.38	12.52
5	68.00	M	OSCC	yes	yes	yes	OSCC - G1	−4.54	−1.94	12.93
6	74.00	F	OSCC	no	no	yes	OSCC - G2	−8.55	−4.53	10.28
7	73.00	M	OSCC	no	no	yes	OSCC - G2	−6.10	−2.10	8.04
8	85.00	M	OSCC	yes	no	yes	OSCC - G2	−3.22	4.27	7.35
9	80.00	F	OSCC	no	no	yes	OSCC - G1	−1.52	11.38	9.46
10	73.00	M	OSCC	no	no	no	OSCC - G2	−7.00	−4.20	10.68
11	52.00	F	OPMD	no	no	yes	high grade dysplasia	3.77	9.75	2.11
12	83.00	M	OPMD	yes	no	no	high grade dysplasia	−8.91	−5.51	2.34
13	60.00	F	OPMD	yes	no	yes	high grade dysplasia	−0.87	1.90	1.61
14	36.00	M	OPMD	yes	no	no	high grade dysplasia	−8.27	−5.50	2.44
15	79.00	M	OPMD	yes	no	yes	high grade dysplasia	−4.03	0.84	3.06
16	61.00	M	OPMD	yes	no	no	high grade dysplasia	3.43	11.57	1.95
17	46.00	M	OPMD	no	no	no	high grade dysplasia	−1.08	7.75	3.18
18	63.00	M	OPMD	yes	no	no	high grade dysplasia	−8.20	−5.83	1.95
19	68.00	M	OPMD	yes	no	yes	high grade dysplasia	2.84	9.87	1.71
20	80.00	F	OPMD	yes	no	yes	high grade dysplasia	6.44	11.38	2.26
21	59.00	M	OPMD	no	no	yes	low-grade dysplasia	−6.59	−2.41	2.87
22	55.00	M	OPMD	yes	no	no	low-grade dysplasia	7.18	12.68	4.52
23	60.00	F	OPMD	no	no	no	low-grade dysplasia	−3.10	−0.24	4.68
24	76.00	F	OPMD	no	no	yes	low-grade dysplasia	−5.62	0.10	5.04
25	78.00	F	OPMD	no	no	yes	low-grade dysplasia	0.62	3.74	6.42
26	60.00	M	OPMD	no	no	yes	low-grade dysplasia	2.12	10.90	4.50
27	60.00	M	OPMD	yes	no	yes	low-grade dysplasia	−5.06	−0.50	4.34
28	53.00	M	OPMD	yes	yes	no	no dysplasia	−5.71	−2.21	5.92
29	51.00	M	OPMD	no	no	yes	no dysplasia	−3.81	0.06	6.30
30	55.00	F	OPMD	no	no	yes	no dysplasia	−5.86	−2.26	11.72
31	53.00	F	OPMD	yes	no	yes	no dysplasia	−3.01	−1.23	6.96
32	52.00	M	OPMD	yes	no	no	no dysplasia	1.45	3.36	13.62
33	68.00	M	OPMD	yes	no	no	no dysplasia	−5.60	−1.52	12.66
34	45.00	M	Controls	yes	no	yes	Controls	−2.67	1.54	4.58
35	23.00	M	Controls	no	no	no	Controls	−3.61	1.34	7.39
36	77.00	M	Controls	no	no	yes	Controls	−5.83	−3.07	11.22
37	67.00	M	Controls	yes	no	no	Controls	−3.70	0.65	2.01
38	75.00	F	Controls	no	no	yes	Controls	−6.72	−3.16	4.38
39	28.00	F	Controls	no	no	no	Controls	−2.21	−0.23	13.59
40	36.00	F	Controls	no	no	no	Controls	−7.05	−4.29	12.32
41	35.00	M	Controls	no	no	no	Controls	−3.60	0.04	9.10
42	29.00	F	Controls	no	no	no	Controls	−3.71	0.16	12.30
43	37.00	M	Controls	no	no	no	Controls	−3.74	−2.66	6.86
